# Dementia among older adults living in long-term care facilities: an epidemiological study

**DOI:** 10.1590/1980-57642021dn15-040007

**Published:** 2021

**Authors:** Daniel Ferreira Fagundes, Marcos Túlio Costa, Bárbara Bispo da Silva Alves, Lara Souza Fernandes Carneiro, Osvaldo Jose Moreira Nascimento, Luana Lemos Leão, André Luiz Sena Guimarães, Alfredo Maurício Batista de Paula, Renato Sobral Monteiro-Junior

**Affiliations:** 1 Centro de Ciências Biológicas e da Saúde, Universidade Estadual de Montes Claros – Montes Claros, MG, Brazil.; 2 Programa de Pós-graduação em Neurologia, Universidade Federal Fluminense – Niterói, RJ, Brazil.; 3 Departamento de Ciências da Educação Física e Desporto, Universidade da Maia – Maia, Portugal.; 4 Centro de Investigação em Desporto, Saúde e Desenvolvimento Humano, Universidade de Trás-os-Montes e Alto Douro – Vila Real, Portugal.; 5 Programa de Pós-graduação em Ciências da Saúde, Universidade Estadual de Montes Claros – Montes Claros, MG, Brazil.

**Keywords:** elderly, homes for the aged, dementia, mild cognitive impairment, idoso, instituição de longa permanência para idosos, demência, disfunção cognitiva

## Abstract

Institutionalization has been associated with social isolation, psychological and cognitive changes, and decreased levels of physical activity in older adults.

**Objectives:**

The aim of this study was to estimate the prevalence of dementia, mild cognitive impairment (MCI), and functional dependence in older adults dwelling in two different Brazilian long-term care facilities (LTCFs).

**Methods:**

This is a cross-sectional study with 185 older people of both sexes, aged 60 years or over, residing in two LTCFs in the city of Montes Claros-MG, Brazil. The diagnosis of MCI and dementia was performed using the *Diagnostic and Statistical Manual of Mental Disorders*.

**Results:**

Prevalence rates of dementia, MCI, and functional dependence in institutionalized older participants were 62.3, 15.1, and 78.9%, respectively. There was a significant reduction of the Mini-Mental State Examination scores according to the increase of the institutionalization period in LCTFs and the age of older adults (p<0.001).

**Conclusions:**

Prevalence of dementia and functional dependence of older adults residing in LTCFs exhibited higher rates compared to the other older population worldwide. A higher institutionalization period is related to a greater cognitive decline.

## RESUMO

A institucionalização tem sido associada ao isolamento social, a alterações psicológicas e cognitivas e à diminuição dos níveis de atividade física em idosos.

**Objetivos:**

Estimar a prevalência de demência, declínio cognitivo leve (DCL) e dependência funcional em idosos residentes em duas instituições de longa permanência (ILPI) brasileiras.

**Métodos:**

Estudo transversal com 185 idosos de ambos os sexos, com 60 anos ou mais, residentes em duas ILPI. O diagnóstico de DCL e demência foi realizado por meio do Manual Diagnóstico e Estatístico de Transtornos Mentais.

**Resultados:**

As taxas de prevalência de demência, DCL e dependência funcional em participantes idosos institucionalizados foram 62,3, 15,1 e 78,9%, respectivamente. Houve redução significativa dos escores do miniexame do estado mental de acordo com o aumento do período de institucionalização nas ILPI e a idade dos idosos (p<0,001).

**Conclusões:**

A prevalência de demência e dependência funcional de idosos residentes em ILPI foi mais elevada em comparação com outras populações idosas em todo o mundo. Um período maior de institucionalização está relacionado a maior declínio cognitivo.

## INTRODUCTION

The number of people with dementia worldwide was estimated to be more than 47 million in 2015. In 2030, it is estimated that there will be more than 75 million people with dementia, reaching 135 million in 2050.^1^ In Brazil, the prevalence of dementia varies from 5.1 to 19% in people aged 60 and over.^2^

With the population aging, family insufficiency and the difficulties of older adults’ caregivers also emerge, increasing the number of individuals residing in long-term care facilities (LTCFs) for older adults.^3^ Among the various reasons that lead older persons to be institutionalized, the limitation to perform activities of daily living (ADL), the onset of cognitive impairment, neurodegenerative diseases, neuropsychological disorders, and caregiver burden are the most common.^4,5^ In addition, institutionalization itself has been associated with social isolation, psychological and cognitive changes, and decreased levels of physical activity in older adults.^6–8^ Furthermore, the decreased general stimuli and family distancing contribute to a possible increase in the prevalence of dementia in LTCFs.^3^ Therefore, this study aimed to estimate the prevalence of dementia, mild cognitive impairment (MCI), and functional dependence in older adults living in Brazilian LTCFs.

## METHODS

### Study design and participants

This is a cross-sectional study conducted from January 2018 to October 2019 following the Strengthening the Reporting of Observational Studies in Epidemiology (STROBE) statement: guidelines for reporting observational studies.^9^

**Figure 1.**
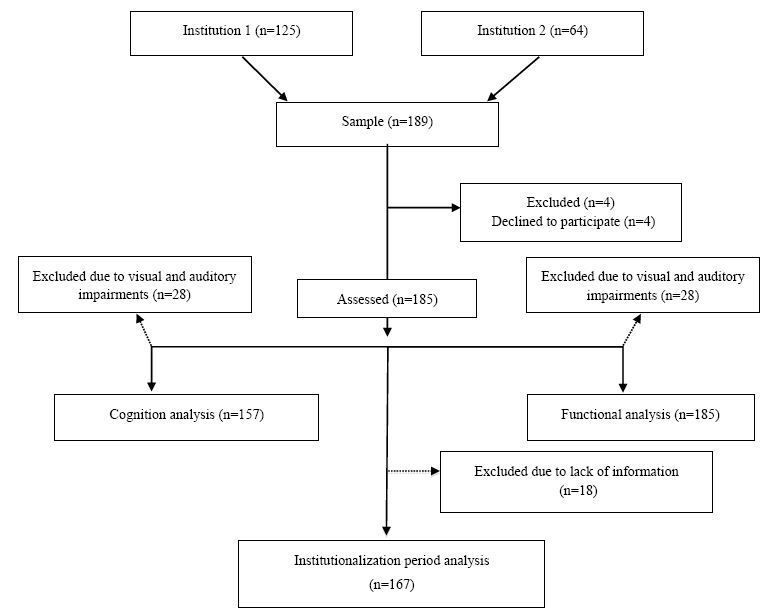
Flowchart displaying the recruitment of participants.

The sample comprised 185 institutionalized older persons from two LTCFs in Montes Claros, Minas Gerais, Brazil (Figure 1). Older men and women aged ≥60 years were recruited. Individuals with severe visual and auditory deficits were excluded from cognitive assessment and determination of the prevalence of dementia. However, these individuals were kept in the study for analysis of functional dependence. The manager of each LCTF signed a consent form on behalf of participants in the present study because most of the institutionalized older adults had the manager as the representative signer. However, participants who were able to sign the consent form were advised to do that. This study was approved by the Research Ethics Committee (No. 2.398.863/2017).

### Cognitive assessment

A neurologist diagnosed MCI and dementia using the *Diagnostic and Statistical Manual of Mental Disorders 5th Edition* (DSM-V).^10^ The Mini-Mental State Examination (MMSE) was used to detect cognitive impairment.^11^ The maximum score is 30 points and the cutoff point is given according to schooling.^12^ MMSE was used to replace Montreal Cognitive Assessment (MoCA). This replacement was chosen due to very low educational level of the participants and to the fact that MoCA presents assessment items that are not part of the routine of institutionalized older adults.

### Functional dependence assessment

The Barthel index was used to assess older adults’ functional dependence. It is an instrument widely used for the assessment of functional independence and mobility.^13^ The Barthel Index assesses basic ADL and measures functional dependence for personal care, mobility, transferences, and eliminations. Each item is scored according to the individual’s performance. An overall score is formed by assigning points to each category. The score ranges from 0 to 100, in five-point intervals, and the higher scores indicate greater independence.^14^ The Barthel index cutoff points are 90–100 independent; 60–89 slightly dependent; 40–59 moderately dependent; 20–39 severely dependent; and <20 completely dependent.^15^

### Data analysis

Descriptive statistics are displayed as measures of central tendency, dispersion, and frequencies. Continuous variables were presented with n, mean, standard deviation, minimum and maximum. Categorical variables were presented with valid absolute and relative frequencies. Data normality was verified through the Kolmogorov-Smirnov test. For each statistical test performed, the conceptual assumptions were verified and assumed. Multiple linear regression and multinomial logistic regression were conducted to identify the associations between continuous and categorical variables, respectively. Nonparametric correlations were also performed through the Spearman test between continuous variables and cross-tabulation with Pearson’s χ^2^ test for categorical variables. All analyses were established at the level p≤0.05, performed in the IBM SPSS^®^ version 24 software.

## RESULTS

Demographic data and sample characterization are shown in Table 1. The sample consisted of a majority of female individuals (56.2%). More than half of women (56.1%) and 40.9% of men were unlettered (Table 1). The prevalence of dementia, MCI, and functional dependence was 62.3, 15.1, and 78.9%, respectively (Table 2). Our sample has shown that 30.3% of institutionalized older adults were completely dependent, while 21.1% were classified as independent. The prevalence of functional dependence and dementia was higher in older adults. The MMSE decreased as the institutionalization period (months) and age (years) increased. Linear regression analysis showed a significant prediction model of 11.8% [F(2.141)=9.402, p<0.001; R^2^=0.118) of the MMSE on the institutionalization period (B=−0.250; t=−3.159; p<0.01; Table 2).

**Table 1.** Sample characterization and demographic data.

	n	Mean	SD	Min	Max
Age (years)	184	79.59	9.64	60	108
Institutionalization (months)	167	78.66	133.36	0	691
MMSE (score)	157	9.78	7.32	0	28
Barthel (score)	185	46.73	35.13	0	100

Level of education	Female		Male		Total
	n	%	n	%	
Unlettered	41	60.3	27	39.7	68
1–7 years	27	44.26	34	55.73	61
≥8 years	5	50	5	50	10
Total	73	52.51	66	47.48	139

MMSE: Mini-Mental State Examination; SD: standard deviation.

**Table 2.** Absolute and relative prevalence of dementia, mild cognitive impairment, and functional dependence.

	n=185		%
Dementia	99		62.3
MCI	24		15.1
Functional dependence	146		78.9
Level of dependence			
Mild dependence	35		18.9
Moderate dependence	25		13.5
Severe dependence	30		16.2
Total dependence	56		30.3

Age	Dementia	MCI	Functional dependence
60–69	51.70%	20.70%	65.60%
70–79	57.10%	21.40%	73.40%
80–89	70.60%	9.80%	85.20%
90–99	66.70%	5.60%	90.90%
100–110	75%	0%	100%

MCI: mild cognitive impairment.

The multinomial logistic regression model showed an association between altered cognition and total functional dependence (OR=6.21; p=0.01, adjusted for sex and age). Although age was significantly associated with total functional dependence (OR=1.07, p=0.01) and moderate functional dependence (OR=1.06, p=0.04), this result is not very significant (Table 3). In addition, women were almost 2.5 times more likely to have dementia than men (OR=2.41; p=0.03).

**Table 3.** Association between functional dependence and impaired cognition.

Level of dependence		OR	p-value	95%CI
Total dependence	Age (years)	1.07	0.01	1.02–1.13
	Sex	0.44	0.09	0.16–1.16
	Impaired cognition	6.21	0.01	1.51–25.53
Severe dependence	Age (years)	1.05	0.11	0.98–1.11
	Sex	0.71	0.53	0.24–2.07
	Impaired cognition	1.13	0.83	0.35–3.60
Moderate dependence	Age (years)	1.06	0.04	1.00–1.13
	Sex	0.68	0.51	0.21–2.16
	Impaired cognition	1.32	0.66	0.36–4.74
Mild dependence	Age (years)	1.05	0.07	0.99–1.11
	Sex	0.47	0.15	0.17–1.31
	Impaired cognition	0.87	0.80	0.29–2.55

OR: *Odds Ratio*; 95%CI: 95% confidence interval; bold values indicate statistical significance.

Language was the MMSE field that most correlated with the overall global cognition score (Rho=0.89, n=146, p<0.01). The best performance in the MMSE was correlated to the best performance in the Barthel index (Rho=0.45, n=157, p<0.01). Temporal orientation was the MMSE field that most correlated with Barthel (Rho=0.39, n=146, p<0.01). Transference was the Barthel item that most correlated with the general functionality score (Rho=0.87, n=174, p<0.01). The variable food was the Barthel index item that most correlated with MMSE (Rho=0.47, n=146, p<0.01).

Older adults with low scores of temporal orientation showed greater functional dependence (χ^2^=14.79; GL=5; p=0.01). Spatial orientation was better in older males (χ^2^=16.36; GL=5; p=0.01). The higher the level of education, the higher the score in spatial orientation (χ^2^=20.4; GL=5; p=0.01). The low score in spatial orientation was associated with greater dependence (χ^2^=14.60; GL=5; p=0.01). Low performance was observed in calculations in older females (χ^2^=14.41; GL=5; p=0.01). There were more dependents in the group with low scores in recent memory (χ^2^=8.1; GL=3; p<0.05) and the change in global cognition was associated with difficulty in intestinal control (χ^2^=6.49; GL=2; p<0.05).

## DISCUSSION

This study identified a 62.3% prevalence of dementia in institutionalized older adults. Furthermore, there was an association between altered cognition and total functional dependence and the institutionalization period. The prevalence of dementia herein shown is greater than that found in a recent meta-analysis,^16^ which showed that 53% of institutionalized older adults were diagnosed with dementia, considering data obtained worldwide. On the other hand, the observed prevalence of dementia in the noninstitutionalized older adults in Brazil ranges from 5.1 to 19%.^2^

Social isolation, loss of contact with the family, low cognitive stimulation, and decreased physical activity contribute to the high prevalence of dementia in LTCFs.^3^ Such data displays a worrying reality, which denotes a high cost for health promotion in LTCFs, in addition to inadequate health, with a propensity to increase the morbidity and mortality rates of institutionalized older adults.

In this study, a low mean of the Barthel index was observed and presented an institutionalized population with many functional impairments. According to the study by Ang et al.,^17^ functional decline is common in LTCFs. The risk factors are age, dementia, and other diseases in institutionalized older adults, such as stroke and urinary incontinence. The level of dependency is a risk factor for older adults to remain institutionalized. Evidence points that cognitive decline affects the functional state.^18^ As noted in this study, the risk of total functional dependence is more than 60 times higher in the older population with impaired cognition. The prevention of functional decline may be possible through better management of dementia, falls, and chronic medical conditions, as well as the implementation of rehabilitation and quality care in LTCFs.^17^

The MMSE decreased according to the institutionalization period, with a decrease of 0.2 points per month (2.4 points per year), which corroborates the study by Harmand et al.,^18^ who found that the institutionalization of older adults was significantly associated with a decrease of 2.8 points in the MMSE score, with a greater increase in cognitive decline after institutionalization and a decrease of 0.2 points per year. Considering that dementia itself is a predictor of institutionalization, older adults with dementia have a higher tendency of being institutionalized than older people with mild dementia/cognitive decline.^19^ Certain components of dementia, such as severity and functional impairment, make older adults more susceptible to institutionalization.^20^ Furthermore, it appears that certain conditions exist in LTCFs, but not yet fully elucidated, which predispose older adults to cognitive decline. Institutionalization could have negative physical and psychological effects on older persons. Another possible element of cognitive decline is the lack of cognitively stimulating activities and physical exercise. In addition, family commitment and participation in social activities, which do not often occur in LTCFs, provide protective effects on older adults’ cognition.^21^ Furthermore, the influence of age on dementia is widely described in the literature and it is known that the prevalence of dementia doubles every 5 years in individuals aged between 65 and 85 years, although these data are from non-institutionalized individuals.^22^

Older women showed an increased risk of dementia compared to male individuals in the present study. According to the study by Zhang et al.,^23^ such a higher prevalence of dementia may be due to longer life expectancy in women. In addition, the higher frequency of low schooling in females may also explain the higher risk of dementia in this group.

Language and food were the fields that most correlated with the mental state of institutionalized older adults, while temporal orientation and transference were the fields that most correlated with the functional profile. Thus, it is assumed that the evaluation of these fields could be used for a simplified screening to check the mental status and functionality of older adults living in LTCFs.

Our study presents some limitations. Other tests, such as MoCA and Pfeffer, are more used to assess cognition and functional dependence in the general older population. However, such instruments do not apply to the reality of LTCFs. Therefore, the MMSE and Barthel index were applied as they provide a more similar approach to the reality of institutionalized older people, although they are not being used as a gold standard for the diagnosis of dementia. The differential diagnosis of the causes of dementia syndromes is difficult to run in LTCFs, due to the absence of companions who deeply know the clinical history of the older adults, the incomplete and not detailed medical records, the lack of complementary exams, and the high frequency of advanced dementia. We limited the diagnosis of people with dementia and non-dementia, without detailing those who have a primary neurodegenerative disease (e.g., Alzheimer’s disease), acquired processes (e.g., stroke), and even potentially reversible diseases (e.g., normal pressure hydrocephalus, neurosyphilis, vitamin deficit, depression, and delirium). Moreover, not having a cognitive assessment at the time of institutionalization makes it difficult to understand how the older adults’ cognition evolves over time in the institution, making it impossible to compare the current cognitive status with cognition on the admission to the LTCF. The majority of Brazilian LTCFs are philanthropic. They receive low or no government support and remain to provide their services supported by donations. These institutions have few workers in their staff and a lack of highly specialized health professionals. However, they resist and survive to keep offering an important service to the community. Additionally, making associations and correlations between variables becomes a challenge, considering the heterogeneous environment of the LTCFs, in which older individuals have different comorbidities, in addition to different social, psychological, and cultural conditions. Finally, subjects were Brazilian citizens, thus belonging to a specific sociocultural context. It this sense, prevents from generalizing results to other countries, characterized by different healthcare and welfare systems, and different family organization.

The prevalence of dementia, MCI, and functional dependence was 62.3, 15.1, and 78.9%, respectively. Institutionalization time might be one of the risk factors for further cognitive decline in older adults. Thus, there is a need for actions at LTCFs, which include measures to improve socialization, cognitive stimulation, physical exercise, rehabilitation, treatment of comorbidities, and adequate management of dementias.
