# Erratum

**DOI:** 10.1590/1980-57642021dn15-040007erratum

**Published:** 2022-01-10

In the manuscript “Dementia among older adults living in long-term care facilities: an epidemiological study”, DOI: 10.1590/1980-57642021dn15-040007 published in the Dement Neuropsychol. 2021;15(4):464-469.

**Where it reads:**

Lara Souza Fernandes Carneiro

**It Should read:**

Lara Carneiro

